# Supplementary material for: Variation in hospital admission in febrile children evaluated at the Emergency Department (ED) in Europe: PERFORM, a multicentre prospective observational study
Source: PLoS One. 2021 Jan 7;16(1):e0244810. doi: 10.1371/journal.pone.0244810 (PMC7790386; doi:10.1371/journal.pone.0244810)
Supplement: S2 Fig — (PDF) [file pone.0244810.s002.pdf]

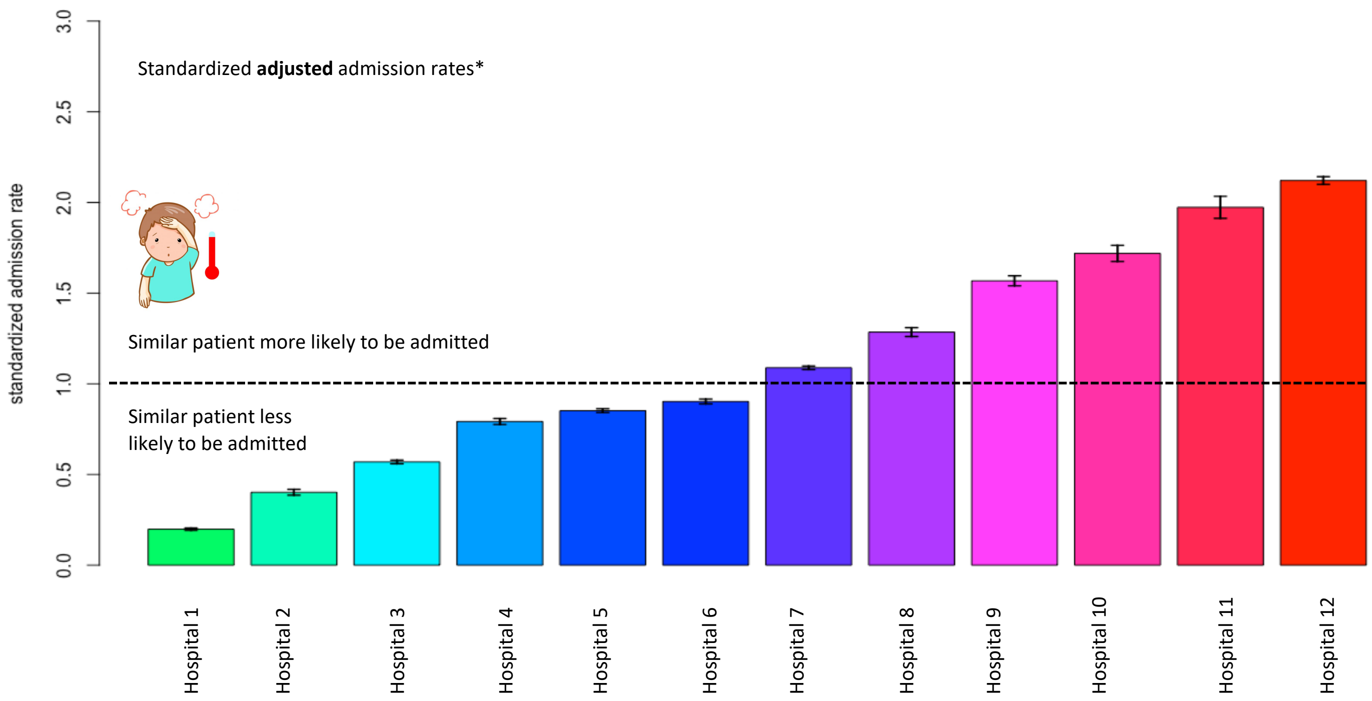

*Multilevel model
- Hospital factors
- General patient factors
- Markers of disease severity
- Diagnostic tests
- Therapy
- Focus / diagnosis
