# Supplementary material for: Variation in hospital admission in febrile children evaluated at the Emergency Department (ED) in Europe: PERFORM, a multicentre prospective observational study
Source: PLoS One. 2021 Jan 7;16(1):e0244810. doi: 10.1371/journal.pone.0244810 (PMC7790386; doi:10.1371/journal.pone.0244810)
Supplement: S1 Table — (PDF) [file pone.0244810.s003.pdf]

Hospital	Country, city	Hospital type	Total annual paediatric ED visits	Total period of inclusion	Period of inclusion per month	Number of patients included	Primary care out-of-office hours	Admission rates
Medizinische Universität Graz	Austria, Graz	University	10,000-30,000	1-1-2017 – 31-12-2018	10 days	2,233	No	21%
Dr. von Hauner Children's Hospital	Germany, Munich	Teaching	10,000-30,000	1-1-2017 – 31-12-2018	1 week	1,172	Yes	10%
P. and A. Kyriakou Children's Hospital	Greece, Athens	University	>30,000	1-1-2017 – 1-5-2018	1-2 weeks	4,525	No	15%
Children clinical university hospital	Latvia, Riga	Teaching	>30,000	1-1-2017 – 31-12-2018	All	8,995	No	28%
Univerzitetni Klinični Center	Slovenia, Ljubljana	University	<10,000	1-1-2017 – 31-12-2018	All	3,649	Yes	55%
Hospital Clínico Universitario	Spain, Santiago de Compostela	University	>30,000	1 Jan 2017 – 1 May 2018	1-2 weeks	3,621	Yes	5%
Erasmus MC-Sophia Children's Hospital	The Netherlands, Rotterdam (NL, 2)	University	<10,000	1 Jan 2017 – 1 Apr 2018	All	1,677	Yes	33%
RadboudUMC	The Netherlands, Nijmegen (NL, 1)	University	<10,000	1 Jan 2017 – 1 Apr 2018	All	674	Yes	45%
Canisius Wilhelmina Ziekenhuis	The Netherlands, Nijmegen (NL, 3)	Teaching	<10,000	1-1-2017 – 31-12-2018	2 weeks	413	Yes	51%
Alder Hey Children's Hospital	United Kingdom, Liverpool (UK, Liv)	Teaching	>30,000	1-1-2017 – 31-12-2018	1 week	1,606	Yes	41%
St. Mary's Hospital	United Kingdom, London (UK, Lon)	University	10,000-30,000	1-1-2017 – 31-12-2018	All	5,712	Yes	22%
Great North Children's Hospital	United Kingdom, Newcastle upon Tyne (UK, New)	University	>30,000	1-4-2017 – 1-4-2018	2 weeks	3,843	Yes	23%
