# Supplementary material for: Variation in hospital admission in febrile children evaluated at the Emergency Department (ED) in Europe: PERFORM, a multicentre prospective observational study
Source: PLoS One. 2021 Jan 7;16(1):e0244810. doi: 10.1371/journal.pone.0244810 (PMC7790386; doi:10.1371/journal.pone.0244810)
Supplement: S2 Table — (PDF) [file pone.0244810.s004.pdf]

Hospital	Any admission N = 9,851	Admission < 24 h N = 2,001	Admission > 24 h N = 7,229	PICU admission N = 156
Austria	1.1 (1.1-1.1)	0.1 (0.1-0.1)	1.6 (1.6-1.6)	2.2 (1.8-.2.6)
Germany	0.7 (0.7-0.7)	0.1 (0.1-0.1)	0.8 (0.8-0.8)	1.1 (0.8-1.4)
Greece	1.5 (1.5-1.5)	0.3 (0.3-0.3)	1.2 (1.2-1.2)	1.7 (1.5-1.9)
Latvia	1.0 (1.0-1.0)	n.a.	1.5 (1.5-1.5)	2.1 (1.9-2.3)
NL, 1	0.9 (0.9-0.9)	1.2 (1.1-1.3)	0.9 (0.9-0.9)	1.8 (1.6-2.0)
NL, 2	0.8 (0.8-0.8)	0.7 (0.7-0.7)	0.8 (0.8-0.8)	0.8 (0.7-0.9)
NL, 3	1.1 (1.1-1.1)	1.7 (1.6-1.8)	1.3 (1.3-1.3)	1.8 (1.4-2.2)
Slovenia	1.2 (1.2-1.2)	1.1 (1.1-1.1)	1.4 (1.4-1.4)	1.6 (1.4-1.8)
Spain	0.6 (0.6-0.6)	1.4 (1.3-1.5)	0.5 (0.5-0.5)	1.4 (1.0-1.8)
UK, Liv	1.3 (1.3-1.3)	5.0 (4.9-5.1)	1.1 (1.1-1.1)	0.2 (0.1-0.3)
UK, New	1.2 (1.2-1.2)	4.7 (4.6-4.8)	0.9 (0.9-0.9)	0.6 (0.5-0.7)
UK, Lon	1.0 (1.0-1.0)	3.1 (3.0-3.2)	0.8 (0.8-0.8)	0.4 (0.4-0.4)

Hospital	Sepsis/meningitis N = 289	URTI* N = 19,947	LRTI** N = 5,621	Fever without focus N = 2,950
Austria	1.0 (0.6-1.4)	1.0 (1.1-1.1)	1.2 (1.2-1.2)	1.0 (0.9-1.1)
Germany	1.0 (0.9-1.1)	0.7 (0.6-0.8)	0.6 (0.6-0.6)	0.8 (0.7-0.9)
Greece	1.0 (0.8-1.2)	1.5 (1.5-1.5)	1.1 (1.1-1.1)	2.7 (2.5-2.9)
Latvia	1.1 (1.0-1.2)	1.1 (1.1-1.1)	1.1 (1.1-1.1)	1.1 (1.1-1.1)
NL, 1	1.0 (0.9-1.1)	0.8 (0.8-0.8)	0.9 (0.8-1.0)	0.9 (0.8-1.0)
NL, 2	1.0 (0.9-1.1)	0.6 (0.6-0.6)	0.8 (0.8-0.8)	0.7 (0.7-0.7)
NL, 3	1.0 (0.8-1.2)	1.0 (0.9-1.1)	1.2 (1.1-1.3)	1.0 (0.9-1.1)
Slovenia	0.9 (0.8-1.0)	1.4 (1.4-1.4)	1.0 (1.0-1.0)	1.1 (1.1-1.1)
Spain	1.0 (0.8-1.2)	0.4 (0.4-0.4)	0.8 (0.8-0.8)	0.5 (0.5-0.5)
UK, Liv	1.0 (0.9-1.1)	1.7 (1.6-1.8)	1.3 (1.3-1.3)	1.1 (1.0-1.2)
UK, New	1.0 (0.9-1.1)	1.5 (1.5-1.5)	1.1 (1.1-1.1)	1.2 (1.1-1.3)
UK, Lon	0.9 (0.8-1.0)	0.8 (0.8-0.8)	1.2 (1.2-1.2)	1.0 (1.0-1.0)

*URTI = upper respiratory tract infection
** LRTI = lower respiratory tract infection

Hospital	Presumed bacterial N = 8,516	Unknown viral/bacterial N = 5,848	Presumed viral N 21,448	Presumed bacterial N = 8,516
Austria	1.3 (1.2-1.3)	1.2 (1.1-1.3)	1.0 (0.9-1.0)	0.9 (0.7-1.0)
Germany	0.8 (0.8-0.9)	0.8 (0.7-0.9)	0.6 (0.6-0.7)	0.3 (0.2-0.4)

**Greece**	1.3 (1.2-1.3)	2.4 (2.3-2.5)	1.2 (1.1-1.2)	2.3 (2.0-2.6)
**Latvia**	0.9 (0.9-0.9)	1.2 (1.2-1.2)	1.1 (1.1-1.1)	0.5 (0.5-0.6)
**NL, 1**	1.0 (0.9-1.0)	0.8 (0.8-0.8)	0.8 (0.8-0.9)	1.1 (1.0-1.2)
**NL, 2**	0.8 (0.8-0.9)	0.8 (0.8-0.8)	0.7 (0.6-0.7)	0.8 (0.8-0.8)
**NL, 3**	1.1 (1.0-1.1)	1.0 (0.9-1.1)	1.1 (1.1-1.2)	0.9 (0.7-1.1)
**Slovenia**	1.0 (1.0-1.0)	1.0 (0.9-1.1)	1.3 (1.3-1.3)	1.2 (1.1-1.3)
**Spain**	0.6 (0.6-0.6)	0.5 (0.4-0.6)	0.6 (0.6-0.7)	0.7 (0.6-0.8)
**UK, Liv**	1.2 (1.2-1.2)	1.5 (1.4-1.6)	1.3 (1.3-1.3)	1.2 (1.2-1.3)
**UK, New**	1.2 (1.1-1.2)	1.2 (1.2-1.2)	1.3 (1.3-1.4)	1.7 (1.6-1.8)
**UK, Lon**	0.8 (0.8-0.9)	1.0 (1.0-1.0)	1.0 (1.0-1.1)	1.1 (1.1-1.2)

Hospital	UTI*<br>N =1,347	Skin/musculoskeletal<br>N = 972	Gastro-intestinal<br>N = 3,958	Exanthems/flulike illness<br>N = 1,866
**Austria**	1.5 (1.4-1.6)	1.3 (1.0-1.6)	0.9 (0.9-0.9)	1.4 (1.3-1.5)
**Germany**	0.7 (0.6-0.8)	0.7 (0.5-0.9)	0.6 (0.5-0.7)	0.8 (0.7-0.9)
**Greece**	1.4 (1.3-1.5)	2.3 (2.1-2.5)	1.6 (1.5-1.7)	2.1 (2.0-2.2)
**Latvia**	1.0 (1.0-1.0)	1.2 (1.0-1.3)	0.8 (0.8-0.8)	1.4 (1.3-1.5)
**NL, 1**	0.8 (0.7-0.9)	1.0 (0.9-1.1)	1.0 (0.9-1.1)	0.3 (0.2-0.4)
**NL, 2**	0.8 (0.7-0.9)	0.7 (0.6-0.8)	0.7 (0.7-0.7)	0.5 (0.5-0.5)
**NL, 3**	1.2 (0.9-1.5)	1.0 (0.9-1.1)	1.2 (1.1-1.3)	n.a.
**Slovenia**	1.0 (1.0-1.0)	0.9 (0.9-0.9	1.2 (1.2-1.2)	1.1 (1.0-1.2)
**Spain**	0.7 (0.6-0.8)	0.8 (0.7-0.9)	0.9 (0.8-1.0)	1.1 (0.9-1.3)
**UK, Liv**	1.5 (1.4-1.6)	1.1 (1.0-1.2)	1.3 (1.2-1.4)	1.9 (1.8-2.0)
**UK, New**	1.2 (1.1-1.3)	1.2 (1.1-1.3)	1.3 (1.2-1.4)	1.8 (1.7-1.9)
**UK, Lon**	0.9 (0.8-1.0)	0.8 (0.8-0.8)	0.7 (0.7-0.7)	1.0 (0.9-1.1)

* UTI = urinary tract infection

Hospital	< 3 months<br>N = 1,049	3-12 months<br>N = 5,722	1-5 years<br>N = 20,460	5-12 years<br>N = 8,338	>12 years<br>N = 2,551
**Austria**	1.2 (1.1-1.3)	1.2 (1.1-1.2)	1.0 (1.0-1.1)	1.0 (0.9-1.0)	1.2 (1.1-1.3)
**Germany**	0.9 (0.8-1.0)	0.7 (0.7-0.8)	0.6 (0.6-0.6)	0.6 (0.6-0.7)	1.0 (0.9-1.2)
**Greece**	1.3 (1.2-1.3)	1.4 (1.3-1.5)	1.5 (1.5-1.5)	1.7 (1.6-1.8)	1.7 (1.5-1.8)
**Latvia**	1.0 (0.9-1.0)	1.1 (1.1-1.1)	1.0 (1.0-1.0)	1.0 (1.0-1.1)	1.0 (1.0-1.1)
**NL, 1**	0.9 (0.9-1.0)	0.9 (0.8-0.9)	0.9 (0.8-0.9)	0.9 (0.9-1.0)	0.9 (0.9-1.0)
**NL, 2**	0.8 (0.8-0.9)	0.7 (0.7-0.8)	0.8 (0.8-0.8)	0.8 (0.8-0.8)	0.7 (0.7-0.7)
**NL, 3**	1.0 (1.0-1.1)	1.1 (1.1-1.2)	1.2 (1.1-1.2)	1.1 (1.0-1.2)	1.0 (0.9-1.1)
**Slovenia**	1.2 (1.2-1.3)	1.2 (1.2-1.2)	1.2 (1.2-1.2)	1.1 (1.1-1.2)	1.1 (1.1-1.1)
**Spain**	0.7 (0.6-0.8)	0.5 (0.5-0.5)	0.6 (0.6-0.6)	0.6 (0.6-0.7)	0.6 (0.5-0.7)
**UK, Liv**	1.2 (1.1-1.2)	1.4 (1.3-1.4)	1.4 (1.4-1.4)	1.2 (1.2-1.3)	1.1 (1.0-1.1)
**UK, New**	0.9 (0.8-0.9)	1.2 (1.1-1.2)	1.3 (1.3-1.3)	1.4 (1.4-1.4)	1.1 (1.0-1.1)
**UK, Lon**	1.0 (0.9-1.0)	0.8 (0.8-0.9)	1.0 (1.0-1.10)	1.0 (1.0-1.0)	1.1 (1.1-1.2)
