# Supplementary material for: Variation in hospital admission in febrile children evaluated at the Emergency Department (ED) in Europe: PERFORM, a multicentre prospective observational study
Source: PLoS One. 2021 Jan 7;16(1):e0244810. doi: 10.1371/journal.pone.0244810 (PMC7790386; doi:10.1371/journal.pone.0244810)
Supplement: S3 Table — * Corrected for patient characteristics, markers of disease severity, diagnostic tests, therapy and working diagnosis. ** UTI = urinary tract infection. (PDF) [file pone.0244810.s005.pdf]

Hospital	UTI	Skin/musculoskeletal	Gastro-intestinal	Exanthems/flulike illness
**Austria**	1.5	1.3	0.9	1.4
**Germany**	0.7	0.7	0.6	0.8
**Greece**	1.4	2.3	1.6	2.1
**Latvia**	1.0	1.2	0.8	1.4
**NL, 1**	0.8	1.0	1.0	0.3
**NL, 2**	0.8	0.7	0.7	0.5
**NL, 3**	1.2	1.0	1.2	0.0
**Slovenia**	1.0	0.9	1.2	1.1
**Spain**	0.7	0.8	0.9	1.1
**UK, Liv**	1.5	1.1	1.3	1.9
**UK, New**	1.2	1.2	1.3	1.8
**UK, Lon**	0.9	0.8	0.7	1.0
