# Supplementary material for: Variation in hospital admission in febrile children evaluated at the Emergency Department (ED) in Europe: PERFORM, a multicentre prospective observational study
Source: PLoS One. 2021 Jan 7;16(1):e0244810. doi: 10.1371/journal.pone.0244810 (PMC7790386; doi:10.1371/journal.pone.0244810)
Supplement: Attachment — Submitted filename: Response to Reviewers.docx [file pone.0244810.s007.docx]

Dear Dr. Chen, dear reviewers

Thank you for your time, effort and expertise in reviewing our manuscript and taking it under consideration for publication.

We have adapted the manuscript according to the comments and we think that the manuscript improved after these important comments.

Please find our response below in blue.

PONE-D-20-27296
Variation in hospital admission in febrile children attending the Emergency Department (ED) in Europe: PERFORM, a multicentre prospective observational study.
PLOS ONE

Dear Dr. Borensztajn,

Thank you for submitting your manuscript to PLOS ONE. After careful consideration, we feel that it has merit but does not fully meet PLOS ONE’s publication criteria as it currently stands. Therefore, we invite you to submit a revised version of the manuscript that addresses the points raised during the review process.

Please submit your revised manuscript by Dec 10 2020 11:59PM. If you will need more time than this to complete your revisions, please reply to this message or contact the journal office at [plosone@plos.org](mailto:plosone@plos.org). When you're ready to submit your revision, log on to <https://www.editorialmanager.com/pone/> and select the 'Submissions Needing Revision' folder to locate your manuscript file.

Please include the following items when submitting your revised manuscript:

- A rebuttal letter that responds to each point raised by the academic editor and reviewer(s). You should upload this letter as a separate file labeled 'Response to Reviewers'.
- A marked-up copy of your manuscript that highlights changes made to the original version. You should upload this as a separate file labeled 'Revised Manuscript with Track Changes'.
- An unmarked version of your revised paper without tracked changes. You should upload this as a separate file labeled 'Manuscript'.

If you would like to make changes to your financial disclosure, please include your updated statement in your cover letter. Guidelines for resubmitting your figure files are available below the reviewer comments at the end of this letter.

If applicable, we recommend that you deposit your laboratory protocols in protocols.io to enhance the reproducibility of your results. Protocols.io assigns your protocol its own identifier (DOI) so that it can be cited independently in the future. For instructions see: [http://journals.plos.org/plosone/s/submission-guidelines#loc-laboratory-protocols](https://eur01.safelinks.protection.outlook.com/?url=http%3A%2F%2Fjournals.plos.org%2Fplosone%2Fs%2Fsubmission-guidelines%23loc-laboratory-protocols&data=04%7C01%7Cd.borensztajn%40erasmusmc.nl%7C19b92504392740a86f9708d879da99da%7C526638ba6af34b0fa532a1a511f4ac80%7C0%7C1%7C637393327137758923%7CUnknown%7CTWFpbGZsb3d8eyJWIjoiMC4wLjAwMDAiLCJQIjoiV2luMzIiLCJBTiI6Ik1haWwiLCJXVCI6Mn0%3D%7C1000&sdata=vKLytWuktOIl9J4VdIRfeW6HAqKokV4rsRcHdqWKmNc%3D&reserved=0)

We look forward to receiving your revised manuscript.

Kind regards,

Tai-Heng Chen, M.D.
Academic Editor
PLOS ONE

Journal Requirements:

When submitting your revision, we need you to address these additional requirements.

1. Please ensure that your manuscript meets PLOS ONE's style requirements, including those for file naming. The PLOS ONE style templates can be found at

<https://journals.plos.org/plosone/s/file?id=wjVg/PLOSOne_formatting_sample_main_body.pdf> and

<https://journals.plos.org/plosone/s/file?id=ba62/PLOSOne_formatting_sample_title_authors_affiliations.pdf>

Thank you for addressing this issue.
We have amended our document to meet PLOS ONE’s style requirements.

2.Thank you for your ethics statement:

'The study was approved by the ethics committees of the participating hospitals. The need for informed consent from individual patients was waived. In all the participating UK settings, an additional opt-out mechanism was in place. Patients were not directly involved in the design of this study.'

Please amend your current ethics statement to include the full name of the ethics committee/institutional review board(s) that approved your specific study.

We have added this information as a separate statement.

*“Ethical approval: The study was approved by the ethical committees of all the participating hospitals and no informed consent was needed for this study. Austria (Ethikkommission Medizinische Universitat Graz, ID: 28-518 ex 15/16), Germany (Ethikkommission Bei Der LMU München, ID: 699-16), Greece (Ethics committee, ID: 9683/18.07.2016), Latvia (Centrala medicinas etikas komiteja, ID: 14.07.201 6. No. Il 16-07 -14), Slovenia (Republic of Slovenia National Medical Ethics Committee, ID: ID: 0120-483/2016-3), Spain (Comité Autonómico de Ética de la Investigación de Galicia, ID: 2016/331), The Netherlands (Commissie Mensgebonden onderzoek, ID: NL58103.091.16), United Kingdom (Ethics Committee, ID: 16/LO/1684, IRAS application no. 209035, Confidentiality advisory group reference: 16/CAG/0136).”*

Once you have amended this/these statement(s) in the Methods section of the manuscript, please add the same text to the “Ethics Statement” field of the submission form (via “Edit Submission”).

We have adjusted the ethics statement in the submission form.

For additional information about PLOS ONE ethical requirements for human subjects research, please refer to <http://journals.plos.org/plosone/s/submission-guidelines#loc-human-subjects-research>.

 3.We note that you have indicated that data from this study are available upon request. PLOS only allows data to be available upon request if there are legal or ethical restrictions on sharing data publicly. For information on unacceptable data access restrictions, please see <http://journals.plos.org/plosone/s/data-availability#loc-unacceptable-data-access-restrictions>.

 In your revised cover letter, please address the following prompts:

 a) If there are ethical or legal restrictions on sharing a de-identified data set, please explain them in detail (e.g., data contain potentially identifying or sensitive patient information) and who has imposed them (e.g., an ethics committee). Please also provide contact information for a data access committee, ethics committee, or other institutional body to which data requests may be sent.

 b) If there are no restrictions, please upload the minimal anonymized data set necessary to replicate your study findings as either Supporting Information files or to a stable, public repository and provide us with the relevant URLs, DOIs, or accession numbers. Please see <http://www.bmj.com/content/340/bmj.c181.long> for guidelines on how to de-identify and prepare clinical data for publication. For a list of acceptable repositories, please see <http://journals.plos.org/plosone/s/data-availability#loc-recommended-repositories>.

Thank you for highlighting this.
We will provide an anonymized data set necessary to replicate our study findings as a public repository with a DOI upon acceptance of our manuscript.
We have added this statement to our cover letter.

We will update your Data Availability statement on your behalf to reflect the information you provide.

4. One of the noted authors is a group or consortium [PERFORM consortium]. In addition to naming the author group, please list the individual authors and affiliations within this group in the acknowledgments section of your manuscript. Please also indicate clearly a lead author for this group along with a contact email address.

 We have added this information to the acknowledgements (S1 acknowledgements).

[Note: HTML markup is below. Please do not edit.]

Reviewers' comments:

Reviewer's Responses to Questions

**Comments to the Author**

1. Is the manuscript technically sound, and do the data support the conclusions?

The manuscript must describe a technically sound piece of scientific research with data that supports the conclusions. Experiments must have been conducted rigorously, with appropriate controls, replication, and sample sizes. The conclusions must be drawn appropriately based on the data presented.

Reviewer #1: Partly

Reviewer #2: Yes

2. Has the statistical analysis been performed appropriately and rigorously?

Reviewer #1: No

Reviewer #2: I Don't Know

3. Have the authors made all data underlying the findings in their manuscript fully available?

The [PLOS Data policy](https://eur01.safelinks.protection.outlook.com/?url=http%3A%2F%2Fwww.plosone.org%2Fstatic%2Fpolicies.action%23sharing&data=04%7C01%7Cd.borensztajn%40erasmusmc.nl%7C19b92504392740a86f9708d879da99da%7C526638ba6af34b0fa532a1a511f4ac80%7C0%7C1%7C637393327137768923%7CUnknown%7CTWFpbGZsb3d8eyJWIjoiMC4wLjAwMDAiLCJQIjoiV2luMzIiLCJBTiI6Ik1haWwiLCJXVCI6Mn0%3D%7C1000&sdata=DK0wteBPmKpqPcoBtuN05mej%2FwGpLXthWi0wq3%2Bvk60%3D&reserved=0) requires authors to make all data underlying the findings described in their manuscript fully available without restriction, with rare exception (please refer to the Data Availability Statement in the manuscript PDF file). The data should be provided as part of the manuscript or its supporting information, or deposited to a public repository. For example, in addition to summary statistics, the data points behind means, medians and variance measures should be available. If there are restrictions on publicly sharing data—e.g. participant privacy or use of data from a third party—those must be specified.

Reviewer #1: Yes

Reviewer #2: Yes

4. Is the manuscript presented in an intelligible fashion and written in standard English?

PLOS ONE does not copyedit accepted manuscripts, so the language in submitted articles must be clear, correct, and unambiguous. Any typographical or grammatical errors should be corrected at revision, so please note any specific errors here.

Reviewer #1: Yes

Reviewer #2: Yes

5. Review Comments to the Author

Please use the space provided to explain your answers to the questions above. You may also include additional comments for the author, including concerns about dual publication, research ethics, or publication ethics. (Please upload your review as an attachment if it exceeds 20,000 characters)

Reviewer #1: 1. It is routine standard of care to admit all febrile children < 1 month, and in some institutions < 2 months. Including these patients may have biased the sample. I suggest the authors separate this group out for a subanalysis.

Thank you for this excellent suggestion. We have added a separate analysis of this age group (table 7) and still found considerable variation between settings.

2. ED admission may also reflect the practice pattern (i.e., conservative vs liberal) of the admitting physician and culture at the hospital site. It also can reflect socioeconomic conditions surrounding each hospital experienced by the patient population. It can also reflect the resources (specialty care, ICU, ID) as stated in the methods description at each hospital. The hospital level factors controlled for are not sufficient to capture those important sources of variation. Therefore, controlling for the 12 hospital clusters in the statistical analysis should be done - you say in your methods you 'analyzed variation between hospitals by age groups, ...' but was it only by those variables?

We apologize for not describing this clearly enough. We have 1) adjusted the admission rates for several confounding variables (hospital level factors, general patient characteristics, markers of disease severity, diagnostic tests and working diagnosis) as described below to compare the different ED’s and 2) performed specific subgroup analyses for age groups, final diagnosis and focus of infection. We adapted the methods section which now reads as follows:

*“We used multilevel logistic regression with a random intercept for each ED to study variation of admission between hospitals. Determinants of admission used in this multilevel logistic regression included hospital-level factors (referral, supervision, primary care availability, regulations on time spent at the ED), general patient characteristics, markers of disease severity, diagnostic tests, therapy and working diagnosis (described in detail in tables 1-3). We included variables if they improved the model defined by a likelihood ratio test using p<0.05 as a cut-off value.*

*We calculated standardised admission rates using indirect standardisation, where the expected number of admissions was standardised to the average ED. Standardised admission ratios are the ratio between observed and expected admissions in an ED. The expected number of admissions was estimated through the adjusted model, by summing the predicted probabilities from the adjusted model of admissions for each patient. Standardised rates >1 demonstrate higher rates than expected and rates <1 indicate lower rates than expected.
We first analysed overall variation between hospitals using the multilevel model described above.
Secondly, we analysed variation between hospitals for specific patient groups, such as age groups, final diagnosis and focus of infection. Standardised rates were visualised in a heat map. [21]”*

3. The conclusions are lacking - it's not sure what these results will add to the practice of emergency medicine, or to health services in general. If it is, as the authors say in the introduction, they wish to describe variation in admission patterns, then looking at variation between the 12 sites would be more illuminating.

*Thank you for pointing out this important issue. We have adjusted the introduction and discussion sections.*

*“We aimed to study variation in hospital admission for febrile children at European EDs, in order to assess whether this variation can be explained by patient characteristics and disease severity, to identify patient groups with remaining large variation after correcting for patient factors, in order to be able to use this data for local hospitals to get insight in potential unnecessary admissions in specific patient groups.”*

*“We found large differences in unadjusted admission rates between the participating EDs. Overall, variation largely diminished after correcting for general patient characteristics, markers of disease severity, diagnostic tests, therapy and focus of infection, showing that, at least in part, the variation in admission rates is related to patient characteristics.
However, variation remained high in specific groups, such as URTI, FWF and the unknown viral/bacterial group.”*

*“However, the fact that we found that for specific subgroups (e.g. upper respiratory tract infections, fever without focus) variation remained high even after correcting for patient characteristics suggests there is a large number of potential unnecessary admissions and there is room for improvement of the management of these patients. This is relevant year-round when in it comes to reducing avoidable health care costs, and even more relevant in peak seasons for hospitalisations, for example due to respiratory syncytial virus.“*

4. Using PEWS is ok, although it is best known for how the change in PEWS value predicts critical illness, rather than one PEWS score snapshot in time. This should be mentioned in the limitations section.

Thank you for addressing this topic. Although, as you mention, the use of PEWS is mainly validated to be used as a change in scores and not a single value and its use in clinical practice at the ED still has to be established, several studies at the ED have shown a single PEWS at arrival at the ED to be associated with (PICU) admission or serious illness.
We have added this statement to the limitations.
Gold, 2014, PMID 25377402; Lillitos 2016, PMID 26531861, Zachariasse 2020, PMID 32710839.

*“Although, the use of PEWS is mainly validated to be used as a change in scores in hospital settings and not a single value. However*, *several studies have shown a single PEWS at arrival at the ED to be associated with general admission, PICU admission and serious illness, [31-33] and as such it was included in our study as a marker of disease severity.”*

5. Why were patients receiving IV antibiotics excluded? This would be a large amount of those admitted, and a reason for admission in itself.

We apologize for this misunderstanding, we only excluded patients where information on intravenous antibiotics were missing, not patients that actually received intravenous antibiotics, we have clarified this in the text.

*“Patients with missing disposition and missing information on intravenous antibiotics were excluded from the analysis”*

6. Not all appropriate pediatric hospital admissions directly relate to disease severity - for example, febrile neonates who appear well and likely have a viral infection, or a cellulitis crossing a major joint - yet those are still appropriate admissions. Therefore, the statements throughout the manuscript that 'overall admission rates reflect disease severity' are not appropriate.

We thank you for highlighting this issue and have changed these statements to “reflect disease severity or the potential risk for a severe disease course.”

Reviewer #2: This is an interesting multicenter paper looking at variation in admission and management practices in febrile children across a network of pediatric EDs in Europe 2017-2018. They describe system differences, illness differences (final diagnosis), testing and other patient differences. The goal of this work is to reduce waste brought upon by unnecessary practice variation. It is timely in that the concern during seasonal flu (and this case COVID 19) has the possibility of really impacting health care resources around Europe and the world.
Some of my suggested edits may be to rewording due to clarity of language (pages refer to pdf #)

Thank you for suggesting improvements regarding language clarity.
We have reworded the statements below according to your suggestions.

p 8 - Title - consider changing attending to evaluation in

*“Variation in hospital admission in febrile children evaluated at the Emergency Department (ED) in Europe: PERFORM, a multicentre prospective observational study.”*

p 12 - Abstract - The comparison of ED admission rates is complex in potentially being influenced by the characteristics of the region, ED, physician and patient. ….in order to use this to reduce health care utilization waste that is often due to practice variation.

“Hospitalisation is frequently used as a marker of disease severity in observational Emergency Department (ED) studies. The comparison of ED admission rates is complex in potentially being influenced by the characteristics of the region, ED, physician and patient. We aimed to study variation in ED admission rates of febrile children, to assess whether variation could be explained by disease severity and to identify patient groups with large variation, in order to use this to reduce unnecessary health care utilization that is often due to practice variation.”

p 14 - the MOFICHE study is a multicentre study evaluating or studying

*“The MOFICHE study is a multicentre study evaluating the management and outcome of febrile children using routinely collected data.”*

- consider adding a sentence about admission as a proxy to severity may be less evidence based when the admission is very short or therapies not escalated.

*“Furthermore, admission rates are commonly used as an outcome measure or proxy for disease severity [11-13] and evidence is needed to assess whether hospitalisation can be used as a valid proxy for disease severity, especially when admission is short or therapy is not escalated.”*

"routine" date - is not a term often used - is there a better one? routinely documented?

Thank you for this suggestion. We have changed the wording into “*routinely collected data”*, which is a term commonly used in research articles.

p 15 - is data collection manual or electronically downloaded?

Thank you for pointing out that this was not described clearly enough. Data were entered into the patient’s record as part of routine care by the treating physician and were then manually extracted from these records and manually entered into an electronic case report form (eCRf) by the research team. We have used your comment to clarify this in the manuscript.

*”Data were entered into the patient’s record as part of routine care by the treating physician and were then manually extracted from these records and manually entered into an electronic case report form (eCRf) by the research team.”*

p 16 - definitions are good, it is not clear if the providers used a case report form for their histories and findings to have standardized data OR if the data was extracted to fit what the provider filled out in the EHR. Please clarify.

Thank you for pointing out that this was not described clearly enough. Data were entered into the patient’s record as part of routine care by the treating physician and were then manually extracted from these records and manually entered into an electronic case report form (eCRf) by the research team. We have used your comment to clarify this in the manuscript.

*”Data were entered into the patient’s record as part of routine care by the treating physician and were then manually extracted from these records and manually entered into an electronic case report form (eCRf) by the research team.”*

p 17 - since you are partly testing diagnostic uncertainty's impact on admission - why not have the clinical diagnosis in the ED and then the discharge diagnosis in the cohort admitted to help understand what evolved?

Thank you for highlighting this point. We collected data on a focus diagnosis that was available during the ED visit and a final diagnosis (viral versus bacterial) that was made after obtaining all test results including cultures, that usually only became available after the ED visit. We only used the information that was available during the ED visit as correction variables in our model (table 3), as test results that were not available to the treating physician, are unlikely to have influenced the treating physician regarding admission or discharge.

p 18 - data quality - not clear if you are training the data extractors OR if you are working to improve the medical records data to prospectively obtain the data you are looking for.

Thank you for pointing out this has not been described clearly enough.
Data quality was improved and standardised by using a digital training module for treating physicians when assessing febrile patients, in order to improve uniform data quality and reduce missing values. For the research team, a standardised eCRF was use.
We have clarified this in the manuscript.

*“Data quality was improved and standardised by using a digital training module for treating physicians at the ED who assess febrile patients, in order to reduce missing values and improve uniform data quality, including the clarification of NICE alarming signs. Clinical data were entered into a standardised eCRF by trained research team members.”*

p 19 - table 1 - was the question of medical care in the past 5 days asked of the patient or linked to actual visits?

The question of medical care in the past 5 days was asked the patients by the treating physician and manually extracted from the patient’s medical records. This included visits at the same facility as well as visits to other facilities.

*“Previous medical care included visits at the same or a different facility and was documented by the treating physician as part of the medical history and manually extracted from the patient’s medical records by the research team.*

Referrals - - if a patient was told to come in by their provider and came by EMS, how is that coded?

Patients referred by a health care provider that were brought in by EMS, were categorised as referral by EMS. We have added this to the manuscript.

*“Patients referred by a health care provider that were brought in by EMS, were categorised as referral by EMS.”*

Vital signs - all patients were febrile or had a history of fever - - did you seek to try and get VS after treatment of the fever to look at change in VS?

Thank you for this excellent suggestion. Unfortunately, we did not collect the data necessary for this analysis.

p 21 - thoughts about putting a figure with the way you derive the standardized vs expected admission in the manuscript? It feels like the concept is important to the work and having a visual regarding it would be good.

Thank you for this important suggestion. We have added a graphic explaining the concept of adjusted standardized admission rates (S4 figure).

The rest of the paper is generally well written and it is fascinating regarding the UK 4-hour rule which may be contributing to the rata of admission and substantial proportion who were in for < 24 hours compared to the other centers.
The ICU admission variation does speak to the question of lack of standardization of ICU admission criteria in pediatrics more broadly, especially since in centers in the US - both the utility of Medical Response Teams (and use of PEWS, for example) may lead to ICU admits that are short and without any or many interventions.

Thank you for these comments. We have emphasized this in the discussion.

*“Additionally, our previous survey among the participating settings of the current study, showed different PICU admission criteria (e.g. the use of high flow oxygen) [15] and a UK study showed a wide variation in PICU admission rates and – similar to our data - highlight the need for clear PICU admission criteria. [25]“*

Just a comment on the provider variability - since there are PEM, pediatricians and both at some hospitals, it seems like a good question of how the amount of experience and the training / perspective of the groups may be important in what your next steps are to get "waste out of the system".

Thank you for addressing this issue. We have added a statement about this to the discussion.

*“Lastly, as previous studies have shown that paediatric emergency physicians are less likely to admit children than general emergency physicians and that the presence of a senior consultant is associated with a reduced rate of total as well as short-stay admissions, training physicians caring for febrile children can play an important part in reducing unnecessary admissions. [22, 44]”*

References - there are a good number of excellent ones

Tables and Figures - Overall these are quite good.

6. PLOS authors have the option to publish the peer review history of their article ([what does this mean?](https://eur01.safelinks.protection.outlook.com/?url=https%3A%2F%2Fjournals.plos.org%2Fplosone%2Fs%2Feditorial-and-peer-review-process%23loc-peer-review-history&data=04%7C01%7Cd.borensztajn%40erasmusmc.nl%7C19b92504392740a86f9708d879da99da%7C526638ba6af34b0fa532a1a511f4ac80%7C0%7C1%7C637393327137768923%7CUnknown%7CTWFpbGZsb3d8eyJWIjoiMC4wLjAwMDAiLCJQIjoiV2luMzIiLCJBTiI6Ik1haWwiLCJXVCI6Mn0%3D%7C1000&sdata=1JMMuQLTD9IlJAbkqoeOArMl2ZKGl3kcyIRTnPzBddo%3D&reserved=0)). If published, this will include your full peer review and any attached files.

If you choose “no”, your identity will remain anonymous but your review may still be made public.

**Do you want your identity to be public for this peer review?** For information about this choice, including consent withdrawal, please see our [Privacy Policy](https://eur01.safelinks.protection.outlook.com/?url=https%3A%2F%2Fwww.plos.org%2Fprivacy-policy&data=04%7C01%7Cd.borensztajn%40erasmusmc.nl%7C19b92504392740a86f9708d879da99da%7C526638ba6af34b0fa532a1a511f4ac80%7C0%7C1%7C637393327137778911%7CUnknown%7CTWFpbGZsb3d8eyJWIjoiMC4wLjAwMDAiLCJQIjoiV2luMzIiLCJBTiI6Ik1haWwiLCJXVCI6Mn0%3D%7C1000&sdata=oibXYFZVOczJyU9fy9Wir11r2k7jQOqDaPIKa1WQCvM%3D&reserved=0).

Reviewer #1: No

Reviewer #2: No

[NOTE: If reviewer comments were submitted as an attachment file, they will be attached to this email and accessible via the submission site. Please log into your account, locate the manuscript record, and check for the action link "View Attachments". If this link does not appear, there are no attachment files.]

While revising your submission, please upload your figure files to the Preflight Analysis and Conversion Engine (PACE) digital diagnostic tool, [https://pacev2.apexcovantage.com/](https://eur01.safelinks.protection.outlook.com/?url=https%3A%2F%2Fpacev2.apexcovantage.com%2F&data=04%7C01%7Cd.borensztajn%40erasmusmc.nl%7C19b92504392740a86f9708d879da99da%7C526638ba6af34b0fa532a1a511f4ac80%7C0%7C1%7C637393327137778911%7CUnknown%7CTWFpbGZsb3d8eyJWIjoiMC4wLjAwMDAiLCJQIjoiV2luMzIiLCJBTiI6Ik1haWwiLCJXVCI6Mn0%3D%7C1000&sdata=wWIzknSFYfVTaO9HJih4F3X684NwshJMABKL1VkleIM%3D&reserved=0). PACE helps ensure that figures meet PLOS requirements. To use PACE, you must first register as a user. Registration is free. Then, login and navigate to the UPLOAD tab, where you will find detailed instructions on how to use the tool. If you encounter any issues or have any questions when using PACE, please email PLOS at [figures@plos.org](mailto:figures@plos.org). Please note that Supporting Information files do not need this step.

*In compliance with data protection regulations, you may request that we remove your personal registration details at any time.* [*(Remove my information/details)*](https://eur01.safelinks.protection.outlook.com/?url=https%3A%2F%2Fwww.editorialmanager.com%2Fpone%2Flogin.asp%3Fa%3Dr&data=04%7C01%7Cd.borensztajn%40erasmusmc.nl%7C19b92504392740a86f9708d879da99da%7C526638ba6af34b0fa532a1a511f4ac80%7C0%7C1%7C637393327137788904%7CUnknown%7CTWFpbGZsb3d8eyJWIjoiMC4wLjAwMDAiLCJQIjoiV2luMzIiLCJBTiI6Ik1haWwiLCJXVCI6Mn0%3D%7C1000&sdata=X7U5nOarTinBqRkMcyrAm2rIAg%2FoBJ2d28Wiue78vUA%3D&reserved=0)*. Please contact the publication office if you have any questions.*
